# Comprehensive Analysis of *SFRP* Family Members Prognostic Value and Immune Infiltration in Gastric Cancer

**DOI:** 10.3390/life11060522

**Published:** 2021-06-03

**Authors:** Dehua Liu, Chenyu Sun, Nahyun Kim, Chandur Bhan, John Pocholo Whitaker Tuason, Yue Chen, Shaodi Ma, Yuting Huang, Ce Cheng, Qin Zhou, Kaiguang Zhang

**Affiliations:** 1 The First Affiliated Hospital of USTC, Division of Life Sciences and Medicine, University of Science and Technology of China, Hefei 230001, China; ldh621@mail.ustc.edu.cn; 2 Internal Medicine, AMITA Health Saint Joseph Hospital Chicago, Chicago, IL 60657, USA; chenyu.sun@amitahealth.org (C.S.); nahyun.kim@amitahealth.org (N.K.); Chandur.bhan@amitahealth.org (C.B.); johnpocholo.tuason@amitahealth.org (J.P.W.T.); 3 Department of Clinical Medicine, School of the First Clinical Medicine, Anhui Medical University, Hefei 230032, China; 1913010067@stu.ahmu.edu.cn; 4 Department of Epidemiology and Health Statistics, School of Public Health Anhui Medical University, Hefei 230032, China; mashaodi@stu.ahmu.edu.cn; 5 University of Maryland Medical Center Midtown Campus, Baltimore, MD 21201, USA; yuting.huang@umm.edu; 6 The University of Arizona College of Medicine, Banner University Medical Center at South Campus, Tucson, AZ 85724, USA; ce.cheng@bannerhealth.com; 7 Banner-University Medical Center South, Tucson, AZ 85713, USA; 8 Radiation Oncology, Mayo Clinic, Rochester, MN 55905, USA; zhou.qin@mayo.edu

**Keywords:** gastric cancer, *SFRPs*, prognostic value, immune infiltration, Wnt pathway

## Abstract

Gastric cancer (GC) is the fifth most common cancer globally. Secreted frizzled-related proteins (SFRP) are important elements associated with the Wnt signaling pathway, and its dysregulated expression is found in multiple cancers. However, the function of distinct *SFRPs* in GC remains poorly understood. We investigated the differential expression, prognostic value, and immune cell infiltration of *SFRPs* in gastric cancer patients from the Oncomine, Gene Expression Profiling Interactive Analysis (GEPIA), UALCAN, Kaplan–Meier plotter, cBioPortal, STRING, Gene-MANIA, DAVID, MethSurv, and TIMER databases. We found that the expression levels of *SFRP2* and *SFRP4* were significantly increased in GC tissues, whereas the *SFRP1* and *SFRP5* expressions were reduced. *SFRP1*, *SFRP2*, and *SFRP5* were significantly correlated with the clinical cancer stage in GC patients. Higher expression of *SFRPs* was associated with short overall survival (OS) in GC patients. Besides, high *SFRPs* methylation showed favorable OS in GC patients. The functions of *SFRPs* were primarily related to the Wnt signaling pathway, immune system development, and basal cell carcinoma. The expression of *SFRPs* was strongly correlated with immune infiltrating cells, including CD4+ T cells and macrophages in GC. Our study indicated that *SFRPs* could be potential targets of precision therapy and prognostic biomarkers for the survival of GC patients.

## 1. Introduction

Gastric cancer (GC) is the fifth most common malignant tumor and is the second leading cause of cancer-associated mortality worldwide according to the GLOBOCAN 2018 estimation [1]. Despite advances in the diagnosis and treatment of cancer, the prognosis of gastric cancer remains unsatisfactory due to the low diagnosis rate, with a 5-year overall survival lower than 40% [2]. Therefore, the exploration of a sensitive and specific biomarker that could predict the prognosis is crucial for GC management.

Secreted frizzled-related proteins (*SFRPs*) are extracellular tumor suppressor genes of Wnt signaling with roles in both embryogenesis and oncogenesis [3]. The loss of SFRP gene expression leading to downstream activation of the Wnt pathway is a vital mechanism for tumorigenesis [4]. In previous reports, frequent promoter hypermethylation and gene silencing of the *SFRPs* were identified in hepatocellular carcinoma and colorectal cancer [5]. Although *SFRPs* have demonstrated potential as effective biomarkers for some cancers, their roles in the development of other tumors are yet to be recognized.

*SFRPs* are modular proteins that contain the signal peptide for secretion followed by a cysteine-rich domain (CRD) [6]. Generally, *SFRPs* are thought to bind directly to Wnt ligands or Frizzled receptors, thereby preventing the initiation of the signaling cascades [7,8]. Some of SFRP genes and proteins have been characterized and studied by common expression profiles, and the aberrant expression of *SFRPs* has been reported to be associated with a variety of cancers [9]. However, the functions and prognostic values of different SFRP family members in GC remain unknown.

In the present study, we mined numerous large databases to analyze the expression, mutation, function, and immune infiltrating of *SFRPs*, with the aim of determining the potential oncogenic and prognostic values of distinct *SFRPs* in GC.

## 2. Materials and Methods

### 2.1. Oncomine

Oncomine (https://www.oncomine.org/resource/login.html, accessed on 27 January 2021) is an integrated online database providing genome-wide expression analysis with cancer microarray information [10]. We used Oncomine database to analyze the expression levels of *SFRPs* family members in different types of cancer. The different mRNA expression levels between GC and normal tissues were analyzed with Student’s *t*-test, with a threshold of *p*-value < 0.01, fold change ≥ 2, and top 10% gene rank.

### 2.2. GEPIA

GEPIA (http://gepia.cancer-pku.cn/, accessed on 28 January 2021) [11] is an interactive web server for analyzing the RNA sequencing expression data from thousands of tumors and normal tissue samples. In our study, GEPIA was utilized to compare differential gene expression between GC and normal tissues. The pathological staging analysis and related prognostic analysis were also performed in GEPIA.

### 2.3. UALCAN

UALCAN (http://ualcan.path.uab.edu/analysis.html, accessed on 26 May 2021) [12] was used to analyze the expression of 5 *SFRPs* genes between GC tissues and corresponding adjoining normal tissues. The difference in transcriptional levels was assessed by students’ *t*-test considering unequal variance, and a *p*-value < 0.05 was considered as statistically significant.

### 2.4. Kaplan–Meier Plotter

Kaplan–Meier plotter (http://www.kmplot.com/, accessed on 27 May 2021) was used to evaluate the prognostic value of *SFRPs* mRNA expression in GC patients [13], which contained the association of gene expression data and survival information of patients with cancer. Hazard ratios (HR) with 95% confidence intervals (CIs) and *p*-values were calculated and displayed in the survival charts, and the *p*-value < 0.05 was considered as statistically significant.

### 2.5. MethSurv

MethSurv (https://biit.cs.ut.ee/methsurv/, accessed on 16 April 2021) is a web tool to perform multivariable survival analysis using Cox proportional risk model, according to the TCGA database [14].

### 2.6. cBioPortal

cBioPortal (http://www.cbioportal.org/, accessed on 29 January 2021) is a comprehensive web resource that provides visualization, analysis, and download of large-scale cancer genomics data sets [15]. In this study, five datasets, namely, “OncoSG 2018”, “TCGA Firehose Legacy”, “Pfizer and UHK Nat Genet 2014”, “UHK Nat Genet 2011”, and “U Tokyo Nat Genet 2014” were used for the analysis of *SFRP* gene mutations.

### 2.7. STRING

STRING (https://string-db.org/, accessed on 27 January 2021) [16] is an online database about predicting protein–protein interactions (10). Different expressions of five *SFRPs* and their possible interactions were collected and integrated through PPI network analysis.

### 2.8. GeneMANIA

GeneMANIA (http://www.genemania.org, accessed on 27 January 2021) [17] was used to predict the protein and genetic interactions, pathways, and functions of five SFRP family members and their related interactors.

### 2.9. DAVID

Functions of *SFRPs* and 20 associated proteins were analyzed by Gene Ontology (GO) and Kyoto Encyclopedia of Genes and Genomes (KEGG) in the DAVID database (https://david.ncifcrf.gov/summary.jsp, accessed on 30 January 2021) [18,19]. GO enrichment analysis could predict the function of *SFRPs* and their 20 related proteins from biological processes (BP), cellular components (CC), and molecular functions (MF), while KEGG analysis could determine the related pathways of *SFRPs* and their associated interactors.

### 2.10. TIMER

TIMER (https://cistrome.shinyapps.io/timer/, accessed on 26 May 2021) is a web resource for assessing the infiltration of different immune cells and their clinical impact [20]. *SFRPs* were input through the “Gene module” and generated plots, and the correlation between their expression and immune infiltration level in gastric cancer was observed.

Additional information of these databases are listed in Supplementary Table S1.

## 3. Results

### 3.1. Differential mRNA Expression Levels of SFRPs in Patients with GC

The transcriptional levels of five *SFRPs* were analyzed in 20 different types of human cancer and compared with normal individuals in the Oncomine database (Supplementary Figure S1). The mRNA expression levels of *SFRP2* and *SFRP4* were significantly elevated in the breast, gastric, and pancreatic cancer tissues, while *SFRP1* showed significantly decreased expression in datasets from 15 different types of cancer. In the Forster Gastric dataset, *SFRP4* over-expression was 8.758-fold higher (*p* = 4.90 × 10^−5^) in diffuse gastric adenocarcinoma tissues than in normal tissues, whereas Cho found a 3.437-fold increase (*p* = 7.77 × 10^−6^) and Chen found a 3.559-fold increase in *SFRP4* mRNA expression (*p* = 1.93 × 10^−18^) in gastric intestinal-type adenocarcinoma tissues (Table 1).

**Figure 1:**
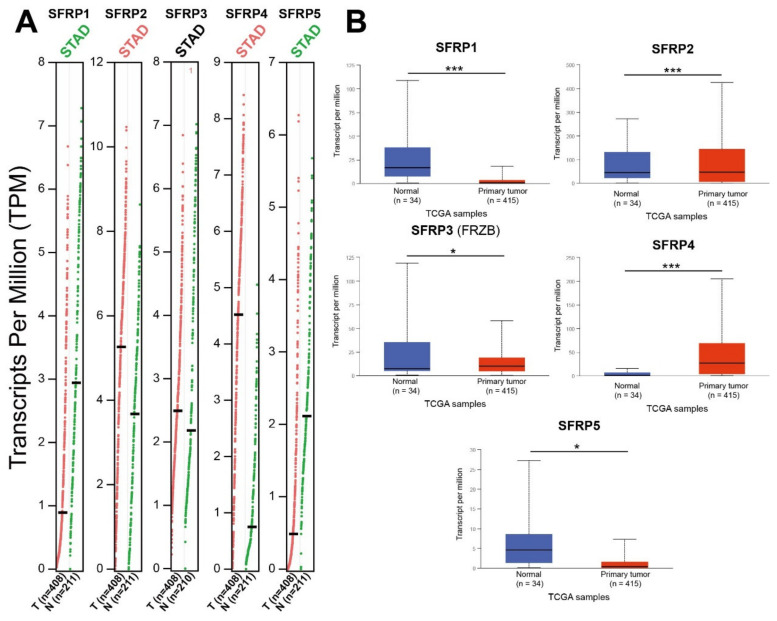
The mRNA expression of distinct *SFRPs* family members in GC tissues and normal gastric tissues (**A**: GEPIA database and **B**: UALCAN database). The method for differential analysis is *t*-test. *: *p* < 0.05 and ***: *p* < 0.001.

We then compared the mRNA expressions of *SFRPs* between GC and normal gastric tissues using the GEPIA dataset. The results showed that the expression levels of *SFRP2* and *SFRP4* in GC tissues were higher than those in normal tissues, and the expression levels of *SFRP1* and *SFRP5* were lower in gastric tissues than in normal tissues. These results were consistent with the findings from the UALCAN data set (Figure 1).

### 3.2. Relationship between SFRPs Expression Levels and Cancer Stages, Subtypes of GC Patients

**Figure 2:**
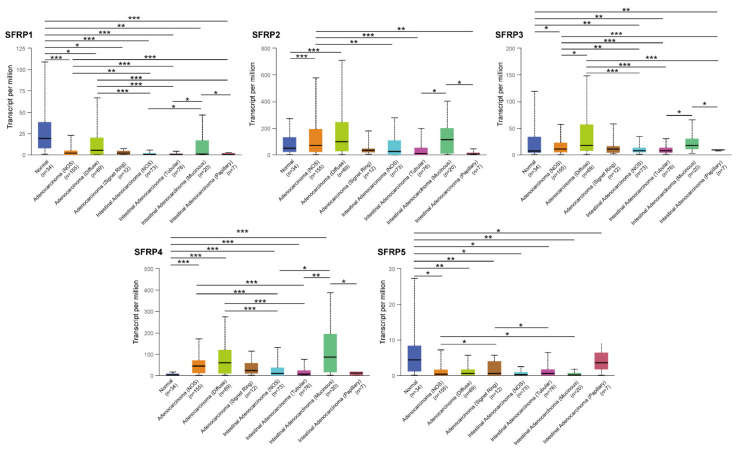
The SFRPs expression in different subtypes of GC (UALCAN database). The method for differential analysis is *t*-test. *: *p* < 0.05, **: *p* < 0.01, and ***: *p* < 0.001. NOS: not otherwise specified.

The expression of *SFRPs* in GC based on histological subtypes was also evaluated (Figure 2). We found that the expression of *SFRP2/3/4* was higher in gastric adenocarcinoma of not otherwise specified (NOS) and diffuse type, and gastric intestinal adenocarcinoma of mucinous type. The expression of *SFRP1/5* was lower in gastric intestinal adenocarcinoma of NOS. We then evaluated the association between *SFRPs* expression and the pathological stage in patients with GC via GEPIA. The expression among tumor stages varied significantly for *SFRP2*, *SFRP3*, and *SFRP4*, whereas the mRNA expressions of *SFRP1* and *SFRP5* were not markedly different (Supplementary Figure S2). We also analyzed the relationship between mRNA expressions of different *SFRPs* family members and individual cancer stages through UALCAN. The mRNA expressions of *SFRP2*/4 were the highest in GC stages 2, 3, and 4. These results suggest that *SFRP2*, *SFRP3*, and *SFRP4* might play an important role in the occurrence and development of GC.

The expression of *SFRPs* in GC based on tumor grade, nodal metastasis status, *TP53* mutation status, and patient’s age was also analyzed and exhibited in Supplementary Figures S3–S6. The differential expressions of *SFRPs* were also found in different subgroups of GC based on tumor grade, nodal metastasis status, and TP53 mutation status. Interestingly, the *SFRP2/3/4* expressions were higher in patients between 41 and 60 years of age.

### 3.3. Prognostic Value of SFRPs mRNA Expression in Patients with GC

**Figure 3:**
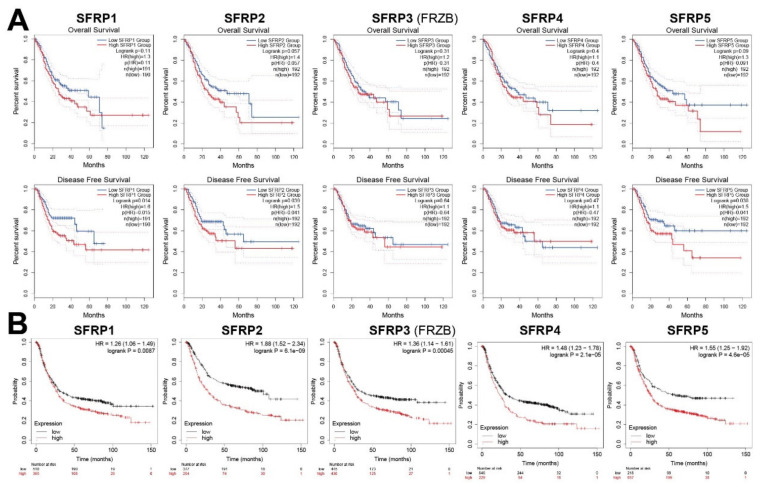
The prognostic value of mRNA level of *SFRPs* in patients with GC. (**A**) GEPIA database and (**B**) Kaplan–Meier plotter. The method for survival analysis is Log-rank test.

To evaluate the value of differential expression of *SFRPs* in GC progression, GEPIA was utilized to analyze the correlation between different *SFRPs* and clinical outcomes. The disease-free survival (DFS) and overall survival (OS) curves showed that GC patients with high transcriptional levels of *SFRP1* (*p* = 0.014), *SFRP2* (*p* = 0.039), and *SFRP5* (*p* = 0.038) were significantly associated with short DFS, but patients with high transcriptional levels of *SFRP3* or *SFRP4* did not show such association (Figure 3A). The Kaplan–Meier plotter was used to analyze the prognostic values of *SFRPs* in patients with GC (Figure 3B). The high mRNA expressions of every *SFRP* family member significantly correlated with short OS in patients with GC (*p* < 0.05). We further evaluated the prognostic values of *SFRPs* in subdivided GC patients based on stages of cancer, Lauren classification, type of treatments, and human epidermal growth factor 2 (HER2) receptor status (Supplementary Figures S7–S10). We found that only high expression of *SFRP1* (*p* < 0.01) correlated with short OS in patients with all stages of GC. In addition, higher expression of *SFRPs* was associated with short OS in intestinal and mixed type GC patients. In surgery-treated and different HER2 status GC patients, the prognostic value of *SFRPs* mRNA expression was consistent.

In addition, we selected the most relevant CpG sites (|*r*| > 0.5, *p* < 0.01) to investigate the prognostic values of *SFRPs* methylation in patients with GC (Supplementary Figure S11). Kaplan–Meier plots demonstrated that low levels of *SFRPs* methylation of the selected CpG sites were correlated with short OS among patients with GC.

### 3.4. Genetic Alteration and Interaction Analyses of SFRPs in Patients with GC

**Figure 4:**
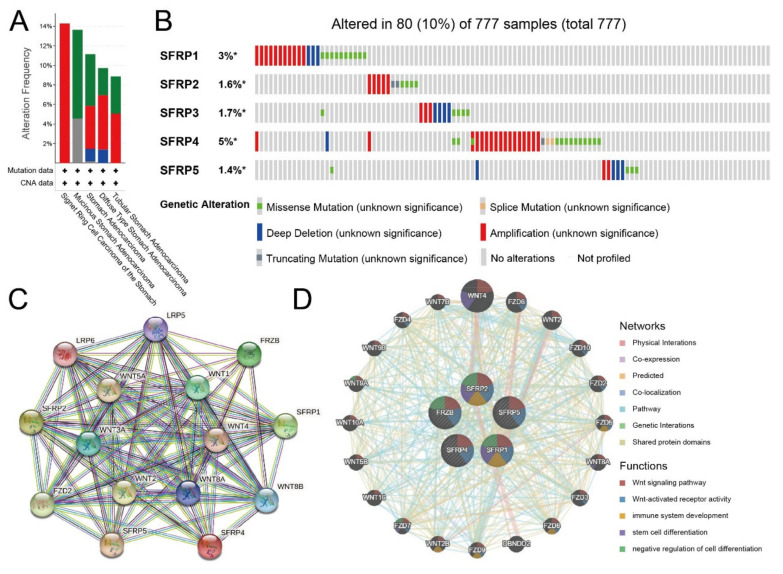
Gene mutation and expression analysis of *SFRPs* in patients with GC (cBioPortal and STRING). (**A**,**B**) Summary of genetic alterations in differently expressed *SFRPs* in GC. (**C**,**D**) Protein–protein interaction network of differently expressed *SFRPs* and their interactors using STRING and GeneMANIA databases, respectively.

Next, we used the cBioPortal online tool to analyze the genetic alterations of *SFRPs* in GC patients. We found that two or more alterations were detected in four subtypes of GC (Figure 4A). Among 777 GC patients sequenced, 80 GC patients had genetic alteration of *SFRPs*, with a mutation rate of 10%. The mutation rates of *SFRP1* and *SFRP4* were the highest, 3% and 5%, respectively (Figure 4B).

The protein–protein interaction (PPI) network analysis was performed on the differentially expressed *SFRPs* and 10 proteins that significantly interacted with *SFRPs* using the STRING database to explore the potential interactions (Figure 4C). These differentially expressed *SFRPs* were associated with regulating the Wnt signaling pathway. The results from GeneMANIA also revealed that the function of differentially expressed *SFRPs* and their associated interactors (such as WNT4, FZD6, WNT2, FZD10, FZD2, FZD5, WNT8A, FZD3, FZD8, and DBNDD2) was primarily related to the Wnt signaling pathway, immune system development, and stem cell differentiation (Figure 4D).

### 3.5. Go Enrichment and KEGG Pathway Analysis of SFRPs

**Figure 5:**
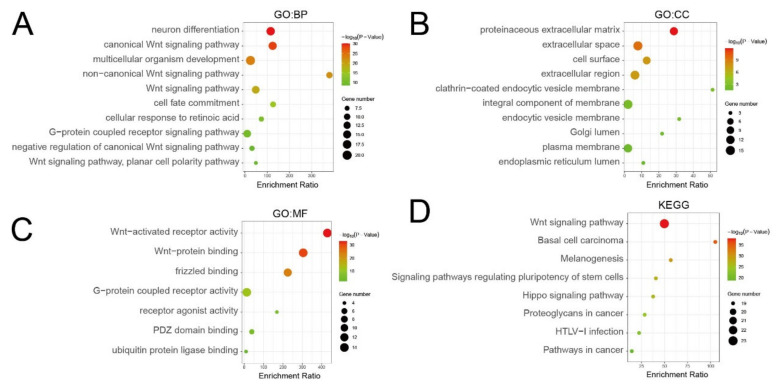
Gene Ontology (GO) enrichment and Kyoto Encyclopedia of Genes and Genomes (KEGG) pathway analysis of *SFRPs* and their interactors (DAVID). GO enrichment analysis of target genes based on (**A**) cellular component, (**B**) biological process, and (**C**) molecular function. (**D**) KEGG pathway enrichment analysis of target genes.

We used DAVID for Go enrichment and KEGG pathway analysis of *SFRPs* and their 20 interactors. The neuron differentiation and the Wnt signaling pathway were the main biological processes that were associated with target genes (Figure 5A). The proteinaceous extracellular matrix was the major cellular component of *SFRPs* and their interactors, and Wnt-activated receptor activity was their primary molecular function (Figure 5B,C). It was found that the Wnt signaling pathway, basal cell carcinoma, and melanogenesis of KEGG pathways for target genes were involved in GC (Figure 5D).

### 3.6. Immune Cell Infiltration of SFRPs in Patients with GC

**Figure 6:**
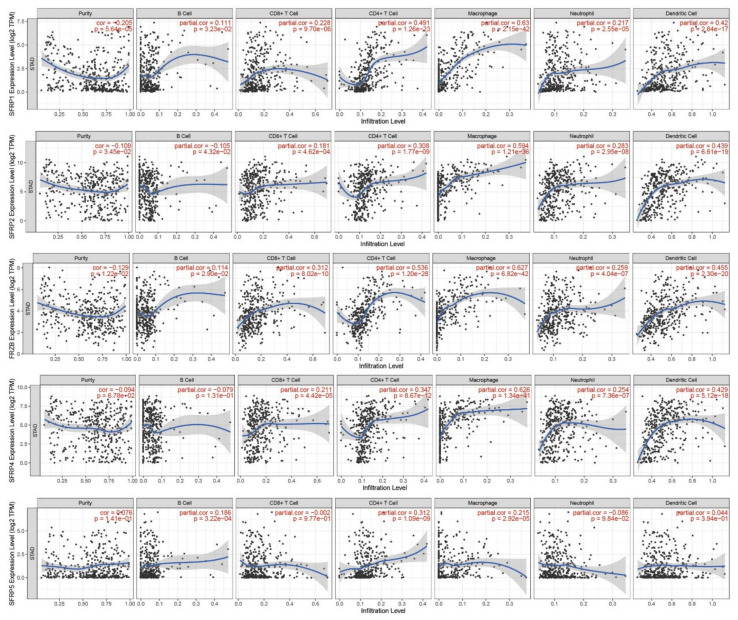
Correlations between differentially expressed *SFRPs* and immune cell infiltration (TIMER). Spearman’s rho value was used for the analysis.

The TIMER database was utilized to investigate the association between *SFRP* family members and immune cell infiltration, as immune cell level correlates with the proliferation and progression of cancer cells (Figure 6). The expressions of *SFRP1/3/4* were positively correlated with the infiltration of CD8+ T cells (*p* < 0.001), CD4+ T cells (*p* < 0.001), macrophages (*p* < 0.001), neutrophils (*p* < 0.001), and dendritic cells (*p* < 0.001). *SFRP2* was positively correlated with the infiltration of CD4+ T cells (*p* < 0.001), macrophages (*p* < 0.001), neutrophils (*p* < 0.001), and dendritic cells (*p* < 0.001). The *SFRP5* expression was positively correlated with the infiltration of CD4+ T cells (*p* < 0.001) and macrophages (*p* < 0.001). In addition, the Cox proportional hazard model showed that CD4+ T cell (*p* = 0.04), macrophage (*p* = 0.013), and *SFRP5* expression (*p* = 0.048) were significantly associated with clinical outcomes in GC patients (Table 2). The Cox proportional hazard model of *SFRPs* and clinical factors in GC was also evaluated (Supplementary Table S2). In addition, the results indicated that the separated *SFRP1/2/4/5* expression, patients’ age, and stage 3/4 were significantly associated with clinical outcomes in GC.

Furthermore, we evaluated the association between *SFRPs* and several gene markers in GC using TIMER (Supplementary Figure S12). We found that the mRNA expressions of *SFRPs* were positively correlated with the expression of *FGFR1* (*p* < 0.001).

## 4. Discussion

Gastric cancer is the fifth most common malignant tumor and the second leading cause of cancer-associated mortality in the world. The pathogenesis of GC is a complex process, which is induced by numerous factors and further stimulated by a variety of pro-oncogenic pathways. The Wnt pathway is involved in important biological processes, such as cell proliferation and differentiation, and abnormal Wnt signaling is commonly observed in several types of cancer [26]. Secreted frizzled-related proteins (*SFRPs*), which are extracellular regulators and tumor suppressors, downregulate Wnt signaling by binding directly to Wnt ligands or Frizzled (Fz) receptors. Present studies have shown that *SFRP* methylation promotes carcinogenesis, especially in hepatocellular carcinoma and colorectal cancer. However, their exact function in GC remains to be elucidated [5]. In our study, we comprehensively analyzed *SFRPs* in terms of expression, mutation, prognostic value, functional enrichment, and immune cell infiltration.

We found that the expression of *SFRP2* and *SFRP4* in GC tissues was higher than that in normal tissues, while the expression of *SFRP1* was decreased in GC tissues in the ONCOMINE database and UALCAN database. The expression of *SFRP5* was also significantly reduced in GC tissues in GEPIA and UALCAN. The expression of *SFRP2*, *SFRP3*, and *SFRP4* in patients with GC significantly correlated with the clinical tumor stage. In addition, the expression of *SFRP2/3/4* was higher in gastric adenocarcinoma of NOS and diffuse type, and gastric intestinal adenocarcinoma of mucinous type. The expression of *SFRP1/5* was lower in gastric intestinal adenocarcinoma of NOS. In addition, the mRNA expressions of *SFRP2/4* were the highest in GC stage 2/3/4, and the *SFRP2/3/4* expressions were higher in patients between 41 and 60 years of age.

Overexpression of *SFRP1*, *SFRP2*, and *SFRP5* in GC significantly correlated with short DFS. Higher expression of *SFRP1/3* correlated with short OS in patients with all stages of GC. We also identified the prognostic values of *SFRPs* in subdivided GC patients based on stages of cancer, Lauren classification, type of treatments, and HER2 status. The higher expression of *SFRPs* was associated with short OS in intestinal and mixed type GC patients, as well as in surgery-treated and different HER2 status GC patients. These results suggest that *SFRPs* are involved in the tumorigenesis of GC and carry potential as a prognostic biomarker for GC.

*SFRP2* has previously been reported as an anti-oncogene whose methylation has been shown to accelerate cancer cell invasion and growth during tumor progression [27]. *SFRP2* can compete with Fz receptors to interact with Wnt proteins via its frizzled-like CRD [6]. A previous study demonstrated that the overexpression of *SFRP2* inhibits the proliferation of oral squamous carcinoma cells and blocks the cell cycle in the G1 phase [28]. The levels of *SFRP1*, *SFRP2*, and *SFRP5* methylation were also reported up-regulated in hepatocellular carcinoma tissues [29]. Previous studies found that the methylation levels of *SFRP2* in gastric carcinoma were lower than adjacent non-cancer samples, and overexpression of *SFRP2* in vivo can inhibit the proliferation of tumor cell and induce cell apoptosis, demonstrated that the methylation of *SFRP2* is an early event in the process of GC [27]. As a result, *SFRP2* can be a novel biomarker and a potential drug target of GC.

Other *SFRPs* are aberrantly expressed in tumors, regulate tumorigenesis, and may serve as potential prognostic biomarkers in GC. *SFRP1* and *SFRP2* have shown oncogenic potential by increasing cellular proliferation or invasion and promoting in vivo tumor growth in renal cancer [30,31]. *SFRP1* was also reported to inhibit several cancers, which was mainly due to epigenetic inactivation via DNA methylation or transcriptional silencing by microRNAs. Epigenetic silencing of *SFRP1* may cause dysregulation of cell proliferation, migration, and invasion [32]. We found that *SFRP1* is significantly reduced in GC tissues and associated with short DFS, thereby warranting further exploration of its functions. The methylation of *SFRP3* promoter was reported frequently in hepatocellular carcinoma [33]. In our study, the expression of *SFRP3* was significantly correlated with gastric cancer stages.

*SFRP4* is a relatively novel Wnt antagonist, which has garnered considerable attention in recent years due to its regulatory action in the Wnt signal transduction system [34]. *SFRP4* is involved in cell proliferation and differentiation and plays an important role in carcinogenesis [35]. Consistent with current literature, we found that the expression of *SFRP4* and gastric cancer stages were positively correlated. A previous finding confirmed the role of *SFRP5* as a physiologic tumor suppressor and demonstrated its potential diagnostic and prognostic value in CRC. We demonstrated that high *SFRP5* expression was significantly correlated with short DFS and OS in GC. Besides, the high *SFRPs* methylation showed favorable OS in GC patients.

In addition, the interaction network and enrichment analysis demonstrated that *SFRPs* and their 20 interactors were mainly associated with the Wnt signaling pathway, immune system development, and basal cell carcinoma. Previous studies showed that *SFRP4* correlates with Treg cell infiltration in pancreatic ductal adenocarcinoma [36]. We further explored the relationship of *SFRPs* and tumor-infiltrating immune cells in GC and found that the expression of *SFRPs* was positively correlated with the infiltration of CD4+ T cells and macrophages. The Cox proportional hazard model indicated that the separated *SFRP1/2/4/5* expression, CD4+ T cell, macrophage, patients’ age, and stage 3/4 were significantly associated with clinical outcomes in GC. In addition, the expression of *SFRPs* was positively correlated with the expression of *FGFR1*, which was an independent prognostic factor in gastric cancer [37]. These findings suggest that *SFRPs* may play a significant role in the tumor microenvironment.

Our study has some limitations that need to be addressed. All the data analyzed in our study came from online databases, and a larger cohort is needed to validate our findings and explore the clinical application of the *SFRPs* members in the GC therapy. In addition, we did not explore the potential mechanisms of distinct *SFRPs* in GC.

## 5. Conclusions

This comprehensive bioinformatics analysis investigated the mRNA expression patterns, prognostic values, genetic alterations, PPI network, functional enrichment, and immune infiltration of *SFRPs* in patients with GC. Our results revealed that *SFRP1*, *2*, and *5* may be new prognostic biomarkers and *SFRP2* *2*, *3*, and *4* may be potential targets for GC. In addition, the high levels of *SFRPs* methylation were associated with better OS among patients with GC. The expression of *SFRPs* correlated significantly with the infiltration of CD4+ T cells and macrophages and the expression of *FGFR1*. Finally, these findings would contribute to novel insights into the distinct roles of *SFRPs* in GC and make a strong argument for further investigation into the application of *SFRP* in GC management.

## Figures and Tables

**Table 1:** The significant changes of *SFRPs* expression in transcription level between gastric cancer and normal gastric tissues (Oncomine).

	Types of GC versus Normal	Fold Change	*p*-Value	*t*-Test	References
*SFRP1*	Diffuse Gastric Adenocarcinoma	2.488	4.79 × 10^−5^	5.003	Chen Gastric [21]
	Diffuse Gastric Adenocarcinoma	12.858	3.47 × 10^−5^	4.54	Forster Gastric [22]
*SFRP2*	Gastric Cancer	9.956	1.78 × 10^−5^	5.019	Wang Gastric [23]
	Diffuse Gastric Adenocarcinoma	10.073	7.00 × 10^−6^	4.982	Forster Gastric [22]
*SFRP3*	Diffuse Gastric Adenocarcinoma	5.896	3.95 × 10^−7^	5.977	Forster Gastric [22]
*SFRP4*	Diffuse Gastric Adenocarcinoma	4.814	5.75 × 10^−10^	8.398	Cho Gastric [24]
	Gastric Intestinal Type Adenocarcinoma	3.437	7.77 × 10^−6^	5.637	Cho Gastric 2 [24]
	Diffuse Gastric Adenocarcinoma	5.36	2.43 × 10^−6^	7.716	Chen Gastric [21]
	Gastric Intestinal Type Adenocarcinoma	3.559	1.93 × 10^−18^	11.327	Chen Gastric 2 [21]
	Gastric Cancer	3.423	3.74 × 10^−7^	4.173	Cui Gastric [25]
	Diffuse Gastric Adenocarcinoma	8.758	4.90 × 10^−5^	4.316	Forster Gastric [22]

SFRP: secreted frizzled-related proteins, GC: gastric cancer, Student’s *t*-test.

**Table 2:** The Cox proportional hazard model of *SFRPs* and six tumor-infiltrating immune cells in GC (TIMER).

	coef	HR	95% CI_l	95% CI_u	*p*-Value	Sig.
B cell	4.041	56.862	0.936	3453.136	0.054	
CD8 T cell	−1.924	0.146	0.009	2.405	0.178	
CD4 T cell	−4.756	0.009	0	0.808	0.04	*
Macrophage	4.655	105.108	2.667	4141.972	0.013	*
Neutrophil	0.608	1.837	0.006	519.318	0.833	
Dendritic	1.055	2.871	0.22	37.5	0.421	
*SFRP1*	−0.057	0.945	0.802	1.112	0.493	
*SFRP2*	0.135	1.145	0.989	1.324	0.07	
*SFRP3*	0.076	1.079	0.901	1.292	0.41	
*SFRP4*	−0.077	0.926	0.796	1.077	0.321	
*SFRP5*	0.112	1.118	1.001	1.249	0.048	*

Coef: coefficient, HR: hazard ratio, CI: confidence interval, sig: significance. * *p* < 0.05.

## Data Availability

The data comes from Oncomine, GEPIA, UALCAN, Kaplan–Meier plotter, cBioPortal, STRING, Gene-MANIA, DAVID, MethSurv, and TIMER databases.

## Supplementary Materials

The following are available online at https://www.mdpi.com/article/10.3390/life11060522/s1, Figure S1: The mRNA expression of *SFRPs* in different cancer types (Oncomine). Difference of transcriptional expression was compared by Students’ *t*-test, Figure S2: Correlations between *SFRPs* expression and tumor stage in GC patients (A: GEPIA database, B: UALCAN database). One-way ANOVA was conducted for differential gene expression analysis using pathological stage as a variable and Log2(TPM + 1) transformed expression data were used for plotting in GEPIA, Figure S3: The *SFRPs* expression in GC based on tumor grade (UALCAN database). The method for differential analysis is *t* test. *: *p* < 0.05, **: *p* < 0.01 and ***: *p* < 0.001. Grade 1: well differentiated, Grade 2: moderately differentiated, Grade 3: poorly differentiated, Grade 4: undifferentiated, Figure S4: The *SFRPs* expression in GC based on nodal metastasis status (UALCAN database). The method for differential analysis is *t* test. *: *p* < 0.05, **: *p* < 0.01, and ***: *p* < 0.001. N0: no regional lymph node metastasis, N1: metastases in 1–3 axillary lymph nodes, N2: metastases in 4–9 axillary lymph nodes, N3: metastases in 10 or more axillary lymph nodes, Figure S5: The *SFRPs* expression in GC based on TP53 mutation status (UALCAN database). The method for differential analysis is *t* test. *: *p* < 0.05, **: *p* < 0.01, and ***: *p* < 0.001, Figure S6: The *SFRPs* expression in GC based on the ages of patients (UALCAN database). The method for differential analysis is *t* test. *: *p* < 0.05, **: *p* < 0.01, and ***: *p* < 0.001. Yrs: years, Figure S7: The prognostic value of mRNA level of *SFRPs* in patients with different GC stages (Kaplan–Meier plotter). The method for survival analysis is Log-rank test, Figure S8: The prognostic value of mRNA level of *SFRPs* in patients with different GC subtypes (Kaplan–Meier plotter). A: intestinal type gastric carcinoma, B: diffuse type gastric carcinoma, C: mixed type gastric carcinoma. The method for survival analysis is Log-rank test, Figure S9: The prognostic value of mRNA level of *SFRPs* in GC patients with different treatment (Kaplan–Meier plotter). A: surgery alone, B: 5 FU-based adjuvant therapy. The method for survival analysis is Log-rank test, Figure S10: The prognostic value of mRNA level of *SFRPs* in GC patients with different HER2 status (Kaplan–Meier plotter). A: HER2 negative and B: HER2 positive. The method for survival analysis is Log-rank test, Figure S11: Kaplan–Meier curves of low and high *SFRPs* DNA promoter CpG sites in GC patients (A: *SFRP1*, B: *SFRP2*, C: *SFRP3*, D: *SFRP4*, and E: *SFRP5*). The method for survival analysis is Log-rank test, Figure S12: Correlations between differentially expressed *SFRPs* and usual gene markers in GC (TIMER). Spearman’s rho value was used for the analysis, Table S1: The information of database, Table S2: The Cox proportional hazard model of *SFRPs* and clinical factors (TIMER).
