# Supplementary material for: Comprehensive Analysis of SFRP Family Members Prognostic Value and Immune Infiltration in Gastric Cancer
Source: Life (Basel). 2021 Jun 3;11(6):522. doi: 10.3390/life11060522 (PMC8228899; doi:10.3390/life11060522)
Supplement: Supplementary file 1 [file life-11-00522-s001.zip › Supplementary-figures.pdf]

Supplementary Materials

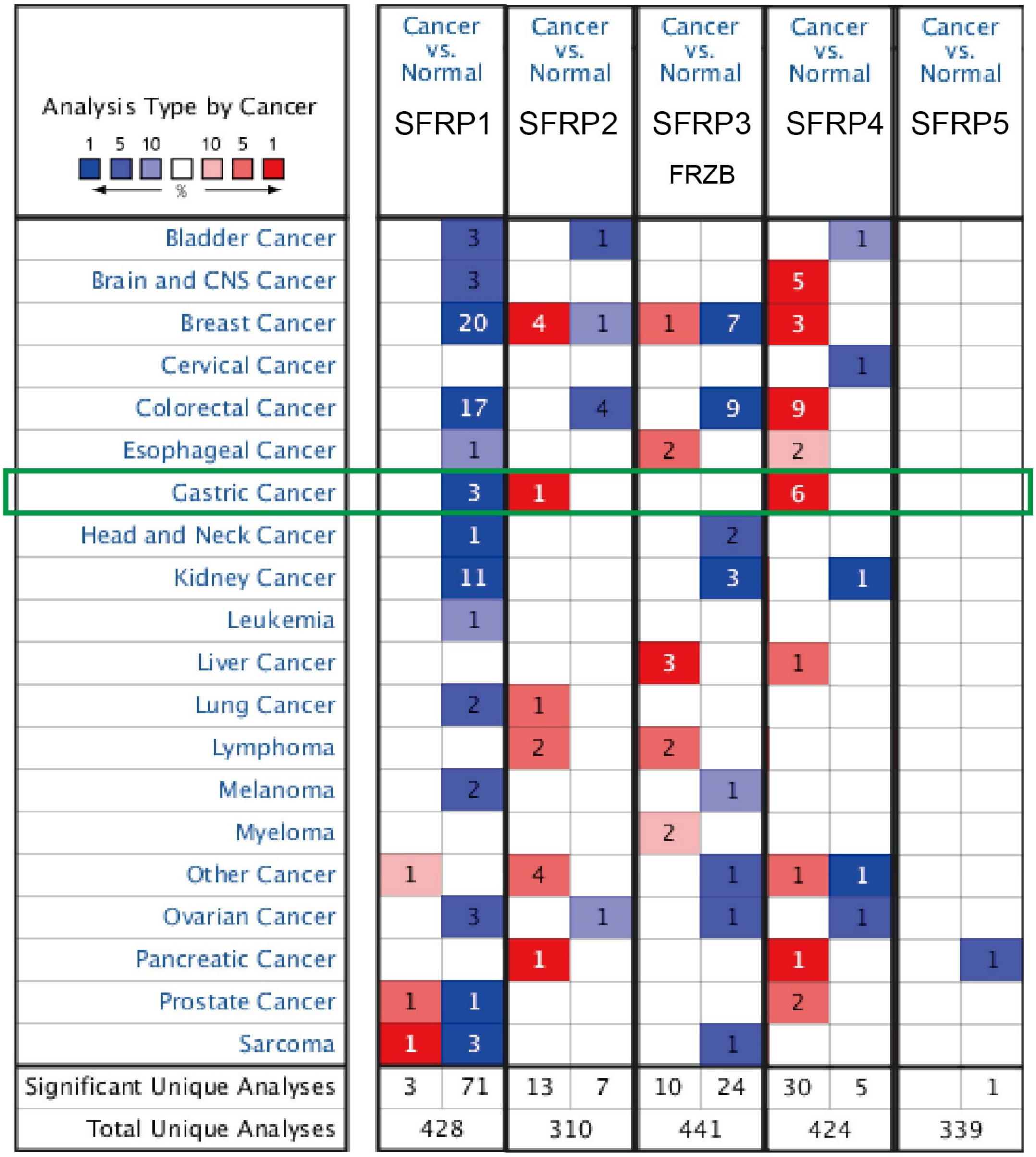

Supplementary Figure S1. The mRNA expression of *SFRPs* in different cancer types (Oncomine). Difference of transcriptional expression was compared by Students' *t*-test.

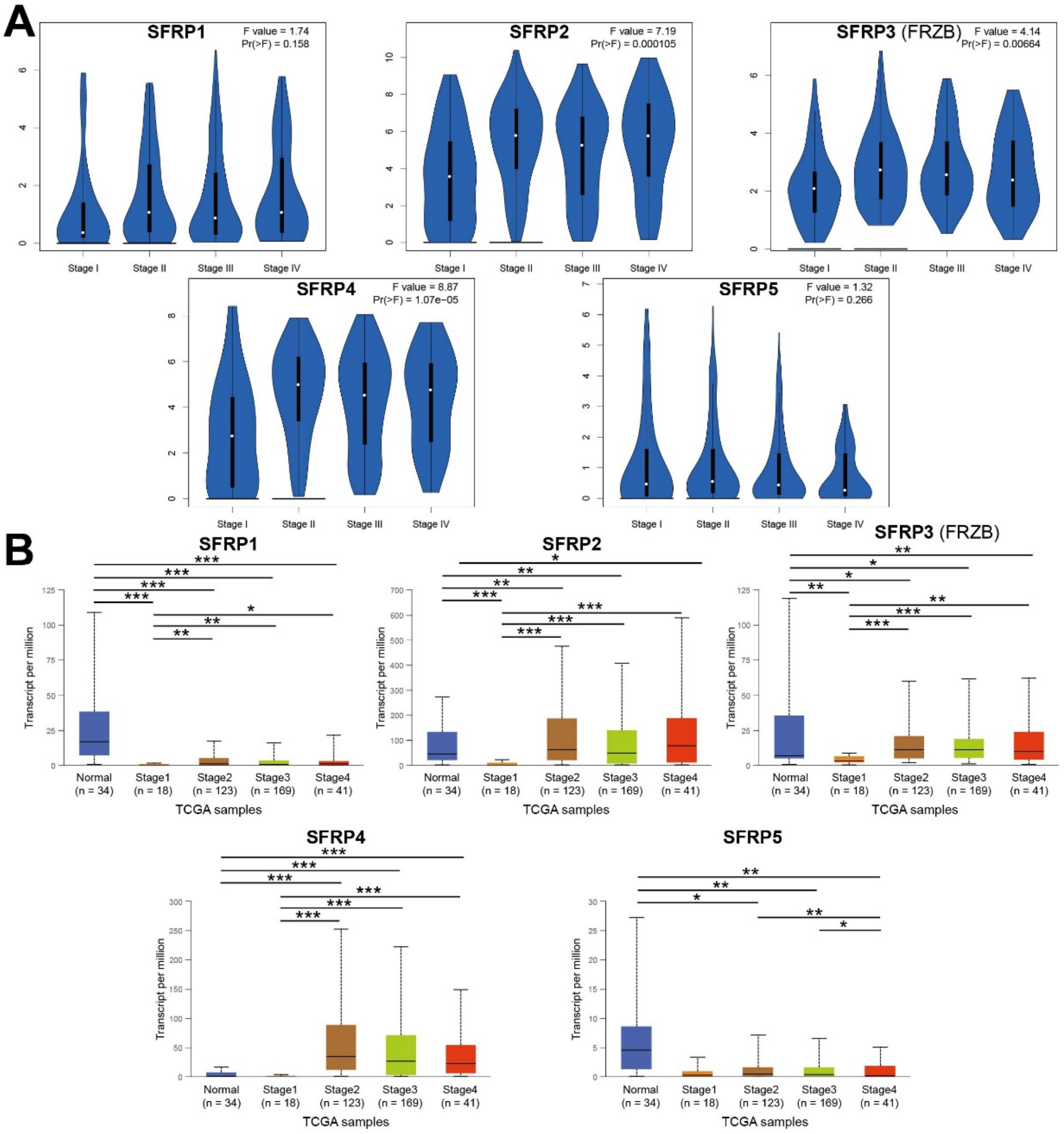

Supplementary Figure S2. Correlations between *SFRPs* expression and tumor stage in GC patients (A: GEPIA database, B: UALCAN database). One-way ANOVA was conducted for differential gene expression analysis using pathological stage as a variable and Log2(TPM + 1) transformed expression data were used for plotting in GEPIA.

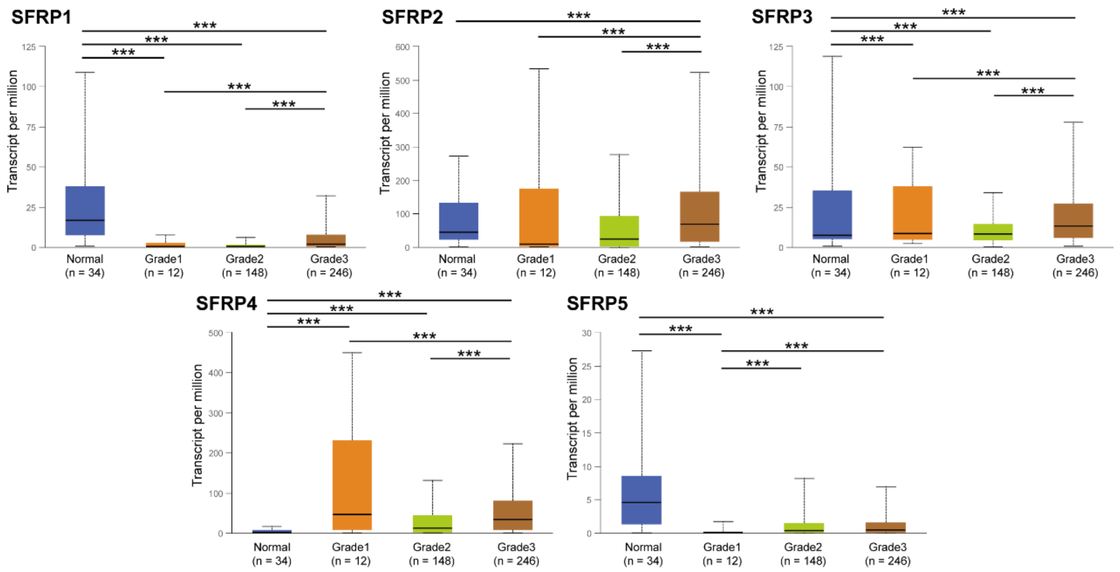

Supplementary Figure S3. The *SFRPs* expression in GC based on tumor grade (UALCAN database). The method for differential analysis is *t* test. *: P<0.05, **: P<0.01, ***: P<0.001. Grade 1: well differentiated, Grade 2: moderately differentiated, Grade 3: poorly differentiated, Grade 4: undifferentiated.

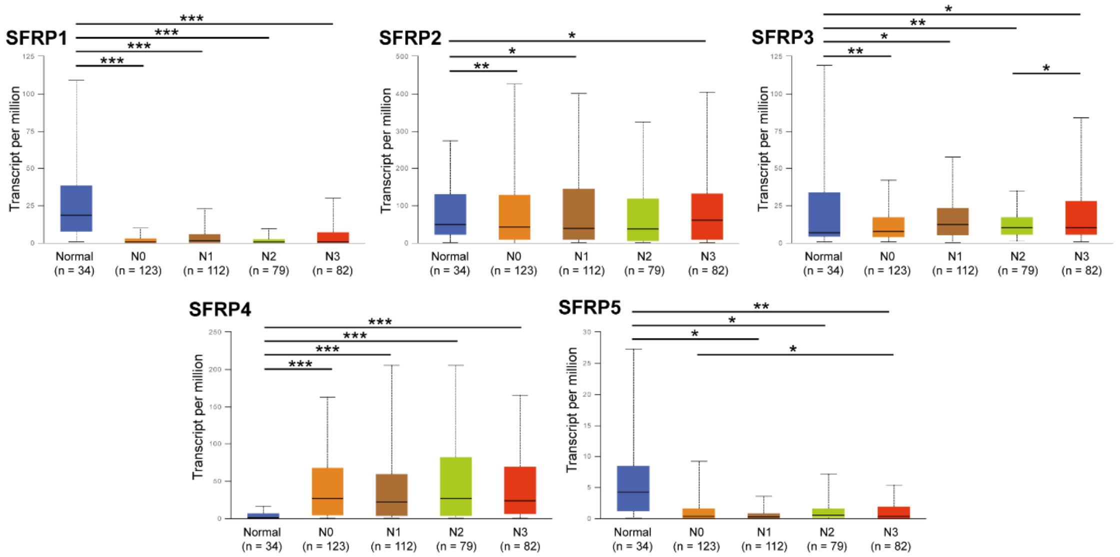

Supplementary Figure S4. The *SFRPs* expression in GC based on nodal metastasis status (UALCAN database). The method for differential analysis is *t* test. *: P<0.05, **: P<0.01, ***: P<0.001. N0: no regional lymph node metastasis, N1: metastases in 1 to 3 axillary lymph nodes, N2: metastases in 4 to 9 axillary lymph nodes, N3: metastases in 10 or more axillary lymph nodes.

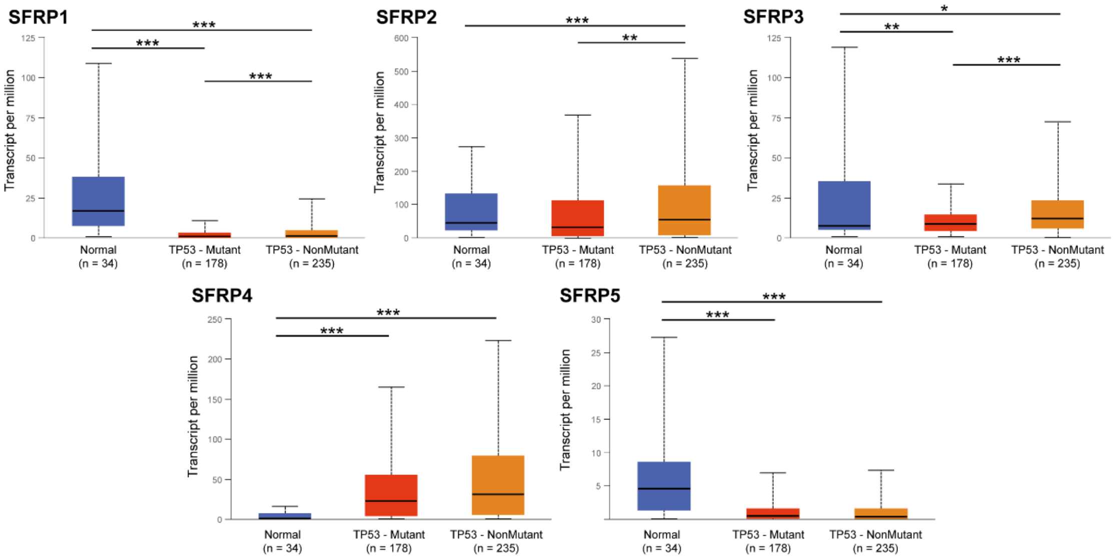

Supplementary Figure S5. The *SFRPs* expression in GC based on TP53 mutation status (UALCAN database). The method for differential analysis is *t* test. *: P<0.05, **: P<0.01, ***: P<0.001.

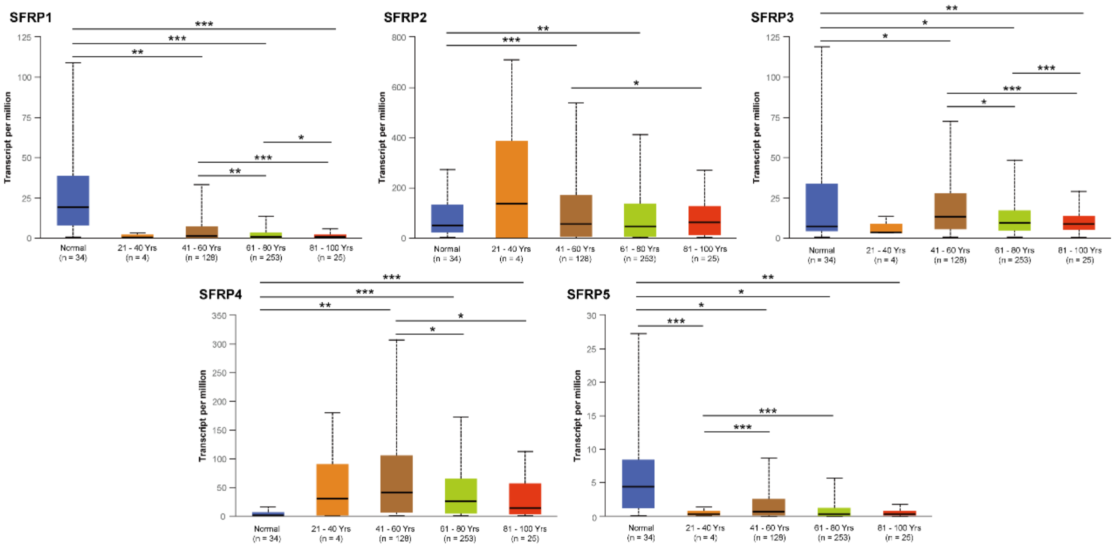

Supplementary Figure S6. The *SFRPs* expression in GC based on the ages of patients (UALCAN database). The method for differential analysis is *t* test. *: P<0.05, **: P<0.01, ***: P<0.001. Yrs: years.

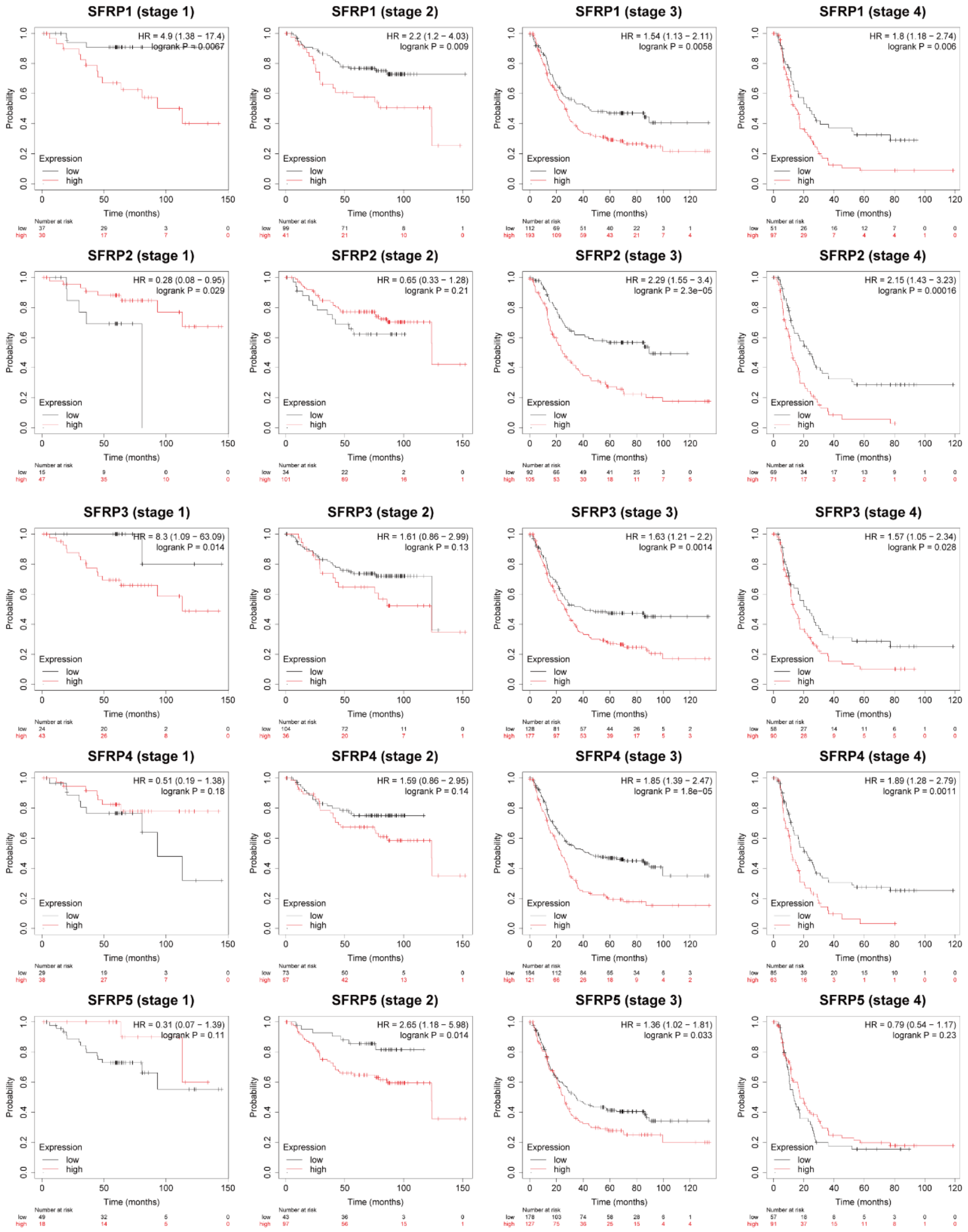

Supplementary Figure S7. The prognostic value of mRNA level of *SFRPs* in patients with different GC stages (Kaplan-Meier Plotter). The method for survival analysis is Log-rank test.

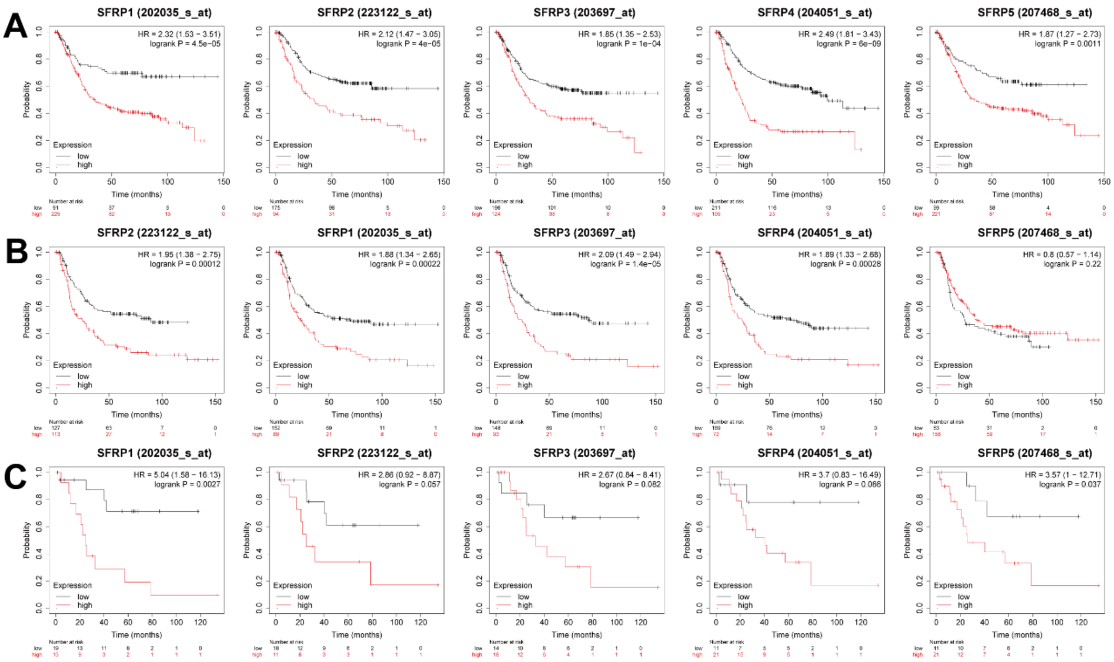

Supplementary Figure S8. The prognostic value of mRNA level of *SFRPs* in patients with different GC subtypes (Kaplan-Meier Plotter). A: intestinal type gastric carcinoma, B: diffuse type gastric carcinoma, C: mixed type gastric carcinoma. The method for survival analysis is Log-rank test.

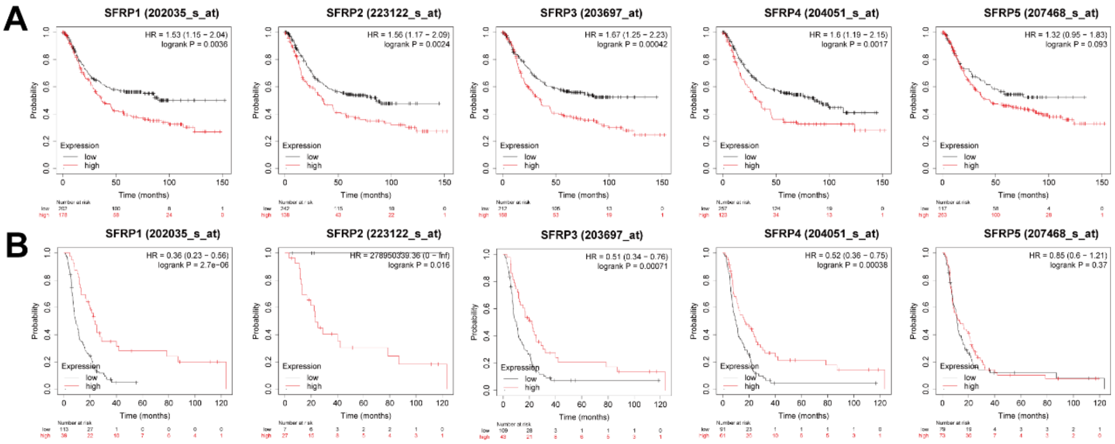

Supplementary Figure S9. The prognostic value of mRNA level of *SFRPs* in GC patients with different treatment (Kaplan-Meier Plotter). A: surgery alone, B: 5 FU based adjuvant therapy. The method for survival analysis is Log-rank test.

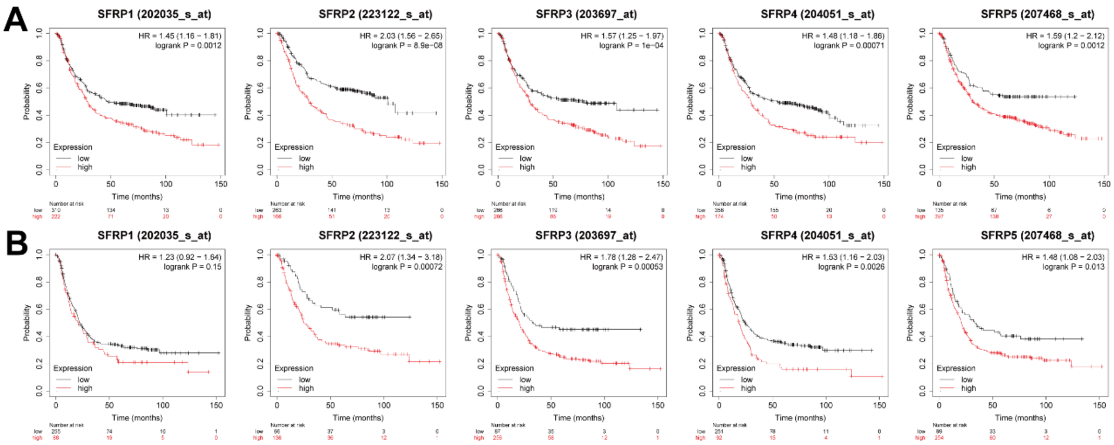

Supplementary Figure S10. The prognostic value of mRNA level of *SFRPs* in GC patients with different HER2 status (Kaplan-Meier Plotter). A: HER2 negative, B: HER2 positive. The method for survival analysis is Log-rank test.

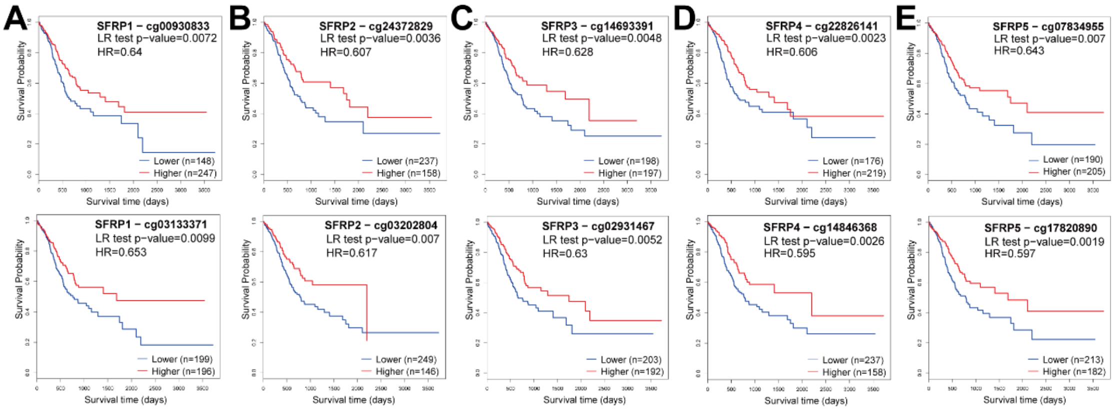

Supplementary Figure S11. Kaplan-Meier curves of low and high *SFRPs* DNA promoter CpG sites in GC patients (A: *SFRP1*, B: *SFRP2*, C: *SFRP3*, D: *SFRP4*, E: *SFRP5*). The method for survival analysis is Log-rank test.

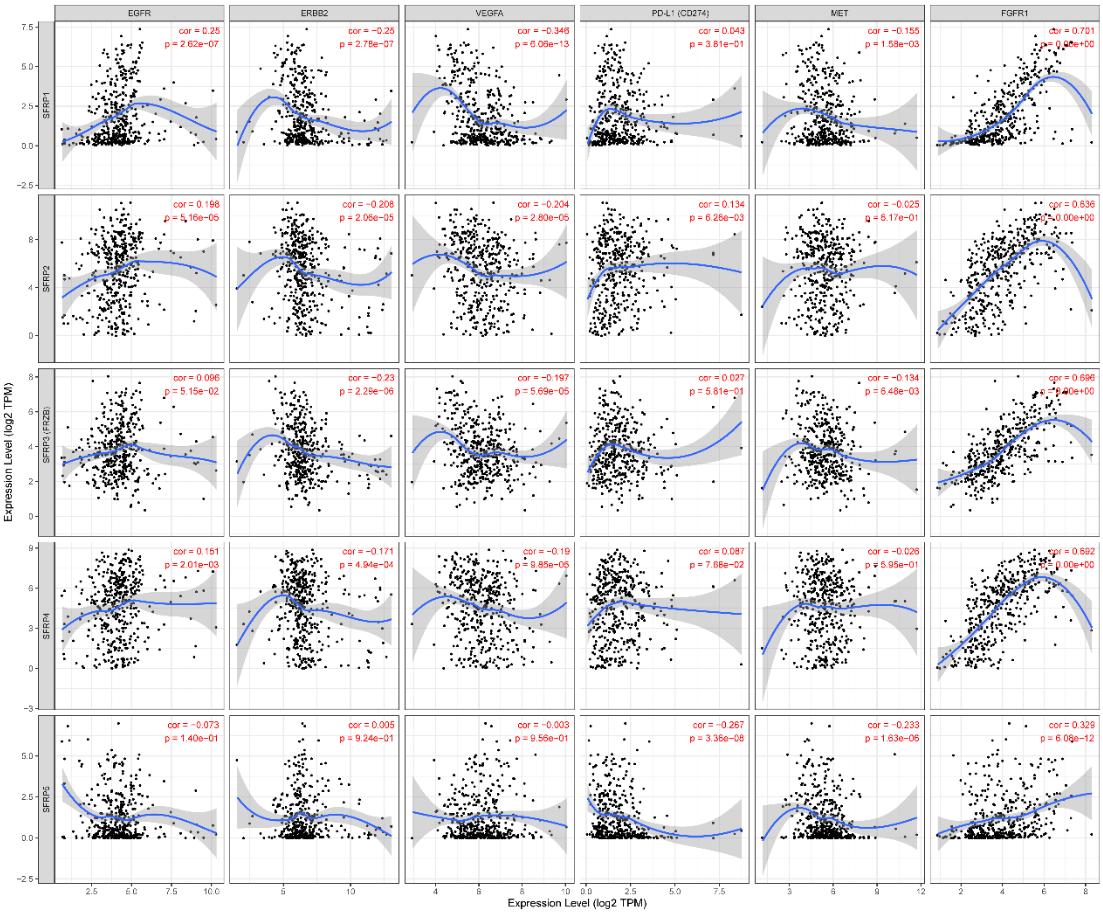

Supplementary Figure S12. Correlations between differentially expressed *SFRPs* and usual gene markers in GC (TIMER). Spearman's rho value was used for the analysis.
